# Paradoxical enrichment of *Akkermansia* in children with poorly controlled asthma: a longitudinal study

**DOI:** 10.3389/fimmu.2026.1807949

**Published:** 2026-05-08

**Authors:** Chian-Feng Huang, Jiu-Yao Wang, Wilfried Joachim Juergen Karmaus, I-Jen Wang

**Affiliations:** 1 Psychiatric Division, Miaoli General Hospital, Ministry of Health and Welfare, Miaoli, Taiwan; 2 Taoyuan Psychiatric Center, Ministry of Health and Welfare, Taoyuan, Taiwan; 3 Allergy, Immunology, and Microbiome (A.I.M.) Research Center, China Medical University Children’s Hospital, Taichung, Taiwan; 4 Division of Epidemiology, Biostatistics, and Environmental Health School of Public Health, University of Memphis, Memphis, MN, United States; 5 Department of Pediatrics, Taipei Hospital, Ministry of Health and Welfare, New Taipei City, Taiwan; 6 School of Medicine, National Yang Ming Chiao Tung University, Taipei, Taiwan; 7 College of Public Health, China Medical University, Taichung, Taiwan; 8 National Institute of Environmental Health Sciences, National Health Research Institutes, Miaoli, Taiwan

**Keywords:** asthma, childhood asthma control test, immunoglobulin E, microbiota, peak expiratory flow

## Abstract

**Introduction:**

Gut dysbiosis is increasingly recognized as a factor in asthma pathogenesis, yet its relationship with disease severity and specific clinical phenotypes remains unclear. This longitudinal study investigated the dynamic changes in gut microbiota composition associated with asthma control and severity in children.

**Methods:**

We identified asthmatic children and matched them with healthy controls within the prospective Taiwan Childhood Environment and Allergic Diseases Cohort Study. Phenotypic data, including childhood asthma control test (C-ACT) scores, clinical severity, serum immunoglobulin E (IgE) levels, and peak expiratory flow (PEF) rates, were collected at the time of fecal sample collection. Gut microbiota composition was assessed using 16S rRNA sequencing and compared between groups. Subgroup analyses and longitudinal paired case comparisons were conducted to track microbial shifts between exacerbation and remission phases.

**Results:**

A total of 173 children, including 82 children with asthma (mean age: 6.9 ± 4.1 years) and 91 age- and gender-matched healthy controls (mean age: 7.5 ± 2.6 years), were recruited. Generally, children with asthma exhibited a lower relative abundance of *Akkermansia*, *Anaerostipes*, and *Escherichia* compared to controls. The relative abundance of *Escherichia* showed a significant negative correlation with C-ACT scores (β = −0.337, p = 0.016), whereas *Akkermansia* exhibited a significant negative correlation with PEF (β = −0.325, p = 0.032). Notably, longitudinal paired case comparisons distinguished asthma attack from remission phases, demonstrating a paradoxical enrichment of *Akkermansia* during exacerbations (LDA = 3.66, p = 0.023).

**Conclusion:**

In contrast to the prevailing view of *Akkermansia* solely as a beneficial microbe, our study identifies a specific severe asthma-associated gut profile characterized by an unexpected enrichment of *Akkermansia* in children with poor control. This finding suggests a complex interaction between the gut microbiome and asthma severity, potentially influenced by high-intensity corticosteroid therapy. These results challenge the one-size-fits-all probiotic approach and highlight the need for precision microbiome-based interventions considering asthma phenotypes and medication history.

## 1 Introduction

Asthma is characterized by reversible airflow obstruction and bronchial hyperresponsiveness, accompanied by underlying inflammation (1). More than 300 million are affected globally (2). It is also the most prevalent chronic respiratory illness among children (3). According to Phase 3 of the International Study of Asthma and Allergies in Childhood (ISAAC), the prevalence of asthma ranges from 3.4% to 31.2% among children aged 13–14 years and from 2.8% to 37.6% among those aged 6–7 years (4). The increasing prevalence of asthma has been paralleled by greater disability and reduced quality of life, which has consequently attracted significant attention from public health professionals.

Allergic asthma, a common phenotype of asthma with early onset in pediatric populations (5), is estimated to affect approximately 38% of asthmatic patients (range, 25%–63%) (6). It is strongly associated with sensitization to diverse allergens, elevated eosinophil counts, and increased levels of immunoglobulin E (IgE), driven by type 2 T-helper cell (Th2)-mediated airway inflammation. The microbial hypothesis (7), posits that reduced beneficial microbial exposure can upregulate Th2 cytokine production, thereby leading to an increase in allergic diseases. Although allergens primarily exert their effects through the respiratory tract, accumulating evidence has highlighted a potential link between gut microbiota composition and asthma development (8).

The human intestinal tract harbors approximately 100 trillion microorganisms (9). The ecology of the gastrointestinal tract evolves from sterility in newborns to a state of adultlike stability by the age of 3 (10). This evolution results in a complex, yet highly regulated system comprising bacteria, fungi, protozoa, and viruses, all of which play crucial roles in maintaining the physiological balance of their host organisms (11). Over 1,500 species across more than 50 phyla have been identified in the human gastrointestinal tract (12). New species of microorganisms and antibiotics may be introduced into the gastrointestinal tract through, for example, the air or diet, and they may disrupt the balance of gut microbiota.

Studies analyzing the gut microbiome of asthmatic children through fecal samples have reported noteworthy alterations. The studies have revealed an increase in the abundance of *Clostridium*, *Escherichia*, *Streptococcus*, and *Staphylococcus* in fecal samples of asthmatic children (13). Furthermore, they have reported a decreased abundance of *Bifidobacterium* and *Lactobacillus* in such samples, suggesting that these bacteria may play a protective role (14). Similarly, high-risk children exhibited decreased levels of *Faecalibacterium*, *Bifidobacterium*, and *Akkermansia* (15).

Microorganisms interact with the microenvironment to modulate local pH, eliminate superoxide radicals, and promote the production of epithelial antimicrobial peptides (16, 17). This intricate interaction extends to the gut mucosal immune system, where microorganisms contribute to increased immunoglobulin A (IgA) secretion, antigen-presenting cell action, and lymphocyte polarization (Th1/Th2) through cytokine regulation (16, 18). In addition, research has discovered that alterations of the microbiome may be linked to the severity of allergic diseases in distant organs (19, 20).

Unlike cross-sectional studies that provide only a static snapshot of the microbiome and primarily report a general depletion or enrichment of certain bacteria, this study aims to elucidate the longitudinal dynamics of the gut microbiota in asthmatic children. By employing a within-subject paired comparison design, we sought to (1): characterize the temporal stability of the gut microbiome across different disease states (exacerbation vs. remission) (2); identify specific microbial signatures that correlate with asthma control levels (C-ACT scores) and severity phenotypes; and (3) evaluate whether these microbial shifts persist after symptom resolution, providing insights into the gut-lung axis mechanisms.

## 2 Materials and methods

### 2.1 Study participants

The study participants were recruited from an outpatient department of a hospital and from the local community as part of the prospective Taiwan Childhood Environment and Allergic Diseases Cohort Study. We identified asthmatic children and matched them with healthy controls. The recruited children and their guardians provided informed consent to participate in this study. The study protocol was approved by the Institutional Review Board of Taipei Hospital, Ministry of Health and Welfare, Taiwan, and adhered to the principles outlined in the Declaration of Helsinki. Asthma was diagnosed in the children on the basis of the criteria established by the Global Initiative for Asthma (GINA). Treatment was given according to GINA guidelines. During acute exacerbations, a short course of systemic oral corticosteroids (Prednisolone equivalent 1 mg/kg/day for 3 days) was administered. Healthy controls were recruited from the community. The inclusion criterion was age < 18 years. Exclusion criteria in both, cases and controls, were an inability to respond to questions in Chinese, prematurity, congenital or severe chronic disease, vegetarianism, the use of probiotics in the past 4 weeks, and the use of antibiotics in the past 4 weeks.

### 2.2 Allergen exposure questionnaire and clinical measurements

All participating children were given the standard version of the ISAAC questionnaire, which included inquiries about environmental allergen exposure. These questionnaires were sent home with the children and were completed by their parents. The survey gathered additional data on demographic characteristics, residential environmental factors (for example, household exposure to tobacco smoke, the presence of pets or cockroaches in the home, house dampness, fungal growth on walls, and the presence of carpets in the home), and the familial history of atopic diseases.

The participants recruited for the asthmatic group were evaluated using clinical measurements, including Childhood Asthma Control Test (C-ACT) scores, Pediatric Asthma Quality of Life Questionnaire scores, Pediatric Asthma Severity scores, peak expiratory flow rates, and medication usage. Some children with asthma completed additional assessments, such as skin prick tests and blood tests. Serum IgE levels were measured through blood tests and were analyzed using enzyme-linked immunosorbent assay.

### 2.3 Fecal sample collection, preparation, and sequencing

Fecal samples were collected using a standard collection method, which involved collection of 5 mL of feces, which were placed in a 50-mL tube and stored at −20 °C immediately after collection. Subsequent total DNA extraction from the fecal samples was conducted using the QIAamp PowerFecal Pro DNA Kit (QIAGEN, Tokyo, Japan) following the manufacturer’s instructions.

The 16S rRNA sequencing libraries were prepared by following the manufacturer’s instructions (Illumina, California, USA). In brief, 12.5 ng of DNA was used for PCR amplification targeting the V3 and V4 regions of the 16S rRNA gene. The amplification was performed using a forward primer (5′-TCGTCGGCAGCGTCAGATGTGTATAAGAGACAGCCTACGGGNGGCWGCAG) and a reverse primer (5′-GTCTCGTGGGCTCGGAGATGTGTATAAGAGACAGGACTACHVGGGTATCTAATCC). After the PCR reaction, the final libraries (approximately 630 bp) were purified using AMPure XP beads (Beckman Coulter, USA).

The prepared libraries underwent paired-end sequencing (2 × 250 bp) on the MiSeq platform (Illumina, California, USA). FASTQ files were generated for subsequent analysis. Taxonomic assignment was performed targeting the V3 and V4 regions based on the Greengenes database (21). Sequence reads were classified at various taxonomic levels including kingdom, phylum, class, order, family, genus, and species.

### 2.4 Statistical and bioinformatics analysis

We analyzed baseline data by using the statistical software package SPSS, version 21.0 (SPSS, Chicago, IL, USA). Two-tailed p values of <0.05 were considered statistically significant. The chi-square test and analysis of variance were applied to identify potential differences in the baseline characteristics between the groups. Following the identification of the relative abundances of various bacterial taxa in each sample, microbial diversity was assessed. Alpha diversity was measured using observed species, the Shannon diversity, and the Chao1 index (22). Beta diversity was analyzed through unweighted and weighed UniFrac distance metrics by using Quantitative Insights Into Microbial Ecology, and principal coordinates analysis (PCoA) plots were generated to compare the bacterial compositions between samples (23, 24). Finally, linear discriminant analysis effect size (LEfSe) was used to identify the taxa that most likely represented the differences between the case and control samples (25). After specifying potential microbial signatures through LEfSe, we selected several specific orders, families, and genera and conducted the Wilcoxon and Kruskal–Wallis tests to identify significant differences in the relative abundances between the asthma and control groups. Similar procedures for longitudinal paired case comparisons between asthma attack and remission phases were also conducted during the follow-up period.

## 3 Results

### 3.1 Baseline characteristics of the study participants

A total of 82 children (mean age: 6.9 ± 4.1 years, 59% male) with asthma were recruited from the outpatient department, and 91 age- and gender-matched healthy controls (mean age: 7.5 ± 2.6 years, 54% male) were recruited from the community. **Table 1** presents a comparison of the baseline characteristics between the 2 groups. The asthma group included more participants with atopic dermatitis and allergic rhinitis, but less participants with smoking environment exposure.

**Table 1:** Baseline characteristics of the participants.

Characteristics	Asthma group (N = 82)	Control group (N = 91)
Age	6.9 ± 4.1	7.5 ± 2.6
Male proportion	59%	54%
BMI	19.3 ± 5.3	18.5 ± 4.3
Atopic Dermatitis*		
Y	27 (32.9%)	13 (14.3%)
N	55 (67.1%)	78 (85.7%)
Allergic Rhinitis*		
Y	59 (72.0%)	33 (36.3%)
N	23 (28.0%)	58 (63.7%)
Food Allergy		
Y	8 (9.8%)	10 (11.0%)
N	69 (84.1%)	81 (89.0%)
Urticaria		
Y	14 (17.1%)	13 (14.3%)
N	68 (82.9%)	78 (85.7%)
Environmental Risk Factors		
Smoking Mother		
Y	6 (7.3%)	4 (4.4%)
N	69 (84.1%)	87 (95.6%)
Smoking Environment (ETS)*		
Y	26 (31.7%)	47 (51.6%)
N	48 (58.5%)	44 (48.4%)
Cockroach exposure at home		
Y	65 (79.3%)	79 (86.8%)
N	15 (18.3%)	12 (13.2%)
Pets at home		
Y	17 (20.7%)	16 (17.6%)
N	62 (75.6%)	75 (82.4%)
Burning incense at home		
Y	42 (51.2%)	41 (45.1%)
N	36 (43.9%)	49 (53.8%)
Mold spot at home		
Y	34 (41.5%)	38 (41.8%)
N	46 (56.1%)	52 (57.1%)

*Significant difference between two groups, p<0.05.

### 3.2 Microbial diversity across asthma severity and phenotypes

**Figure 1:**
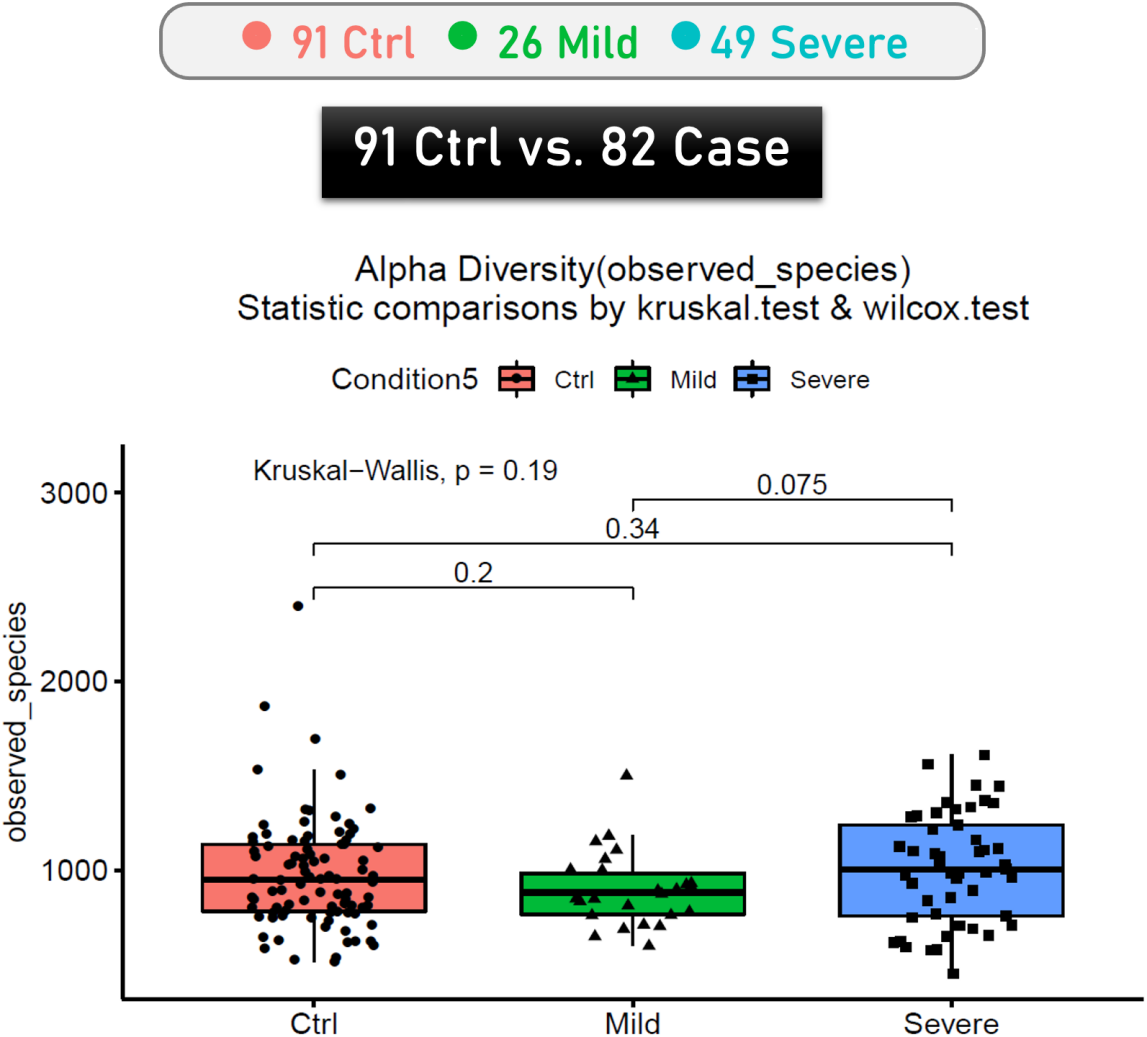
Comparison of alpha diversity among severe asthma, mild asthma and control groups based on observed species richness.

**Figure 2:**
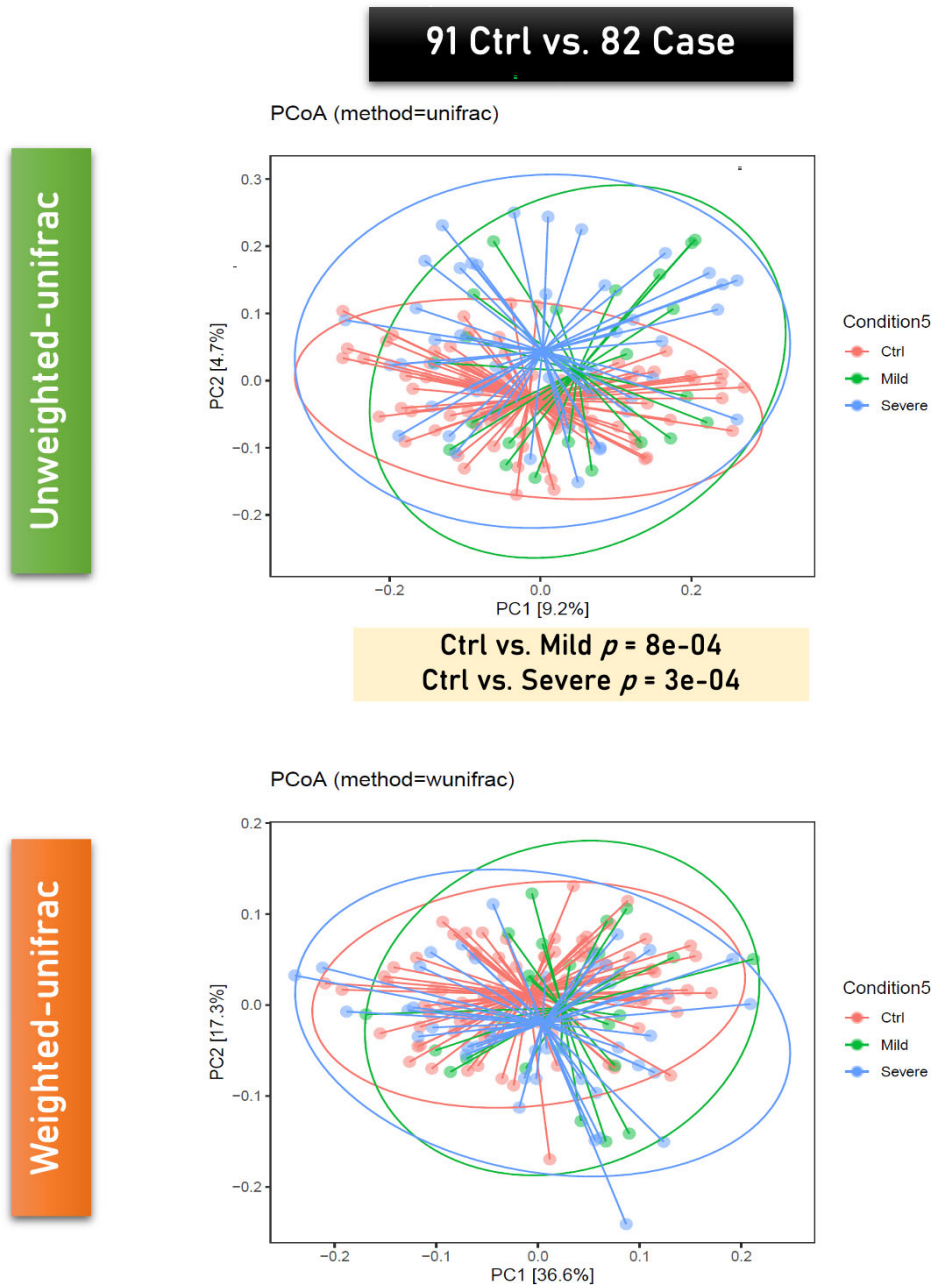
Comparison of beta diversity among severe asthma, mild asthma and control groups conducted using principal coordinate analysis (PCoA). Upper panel, unweighted UniFrac; lower panel, weighted UniFrac.

Based on the four symptom-based severity categories defined by the Global Initiative for Asthma (GINA) guidelines, we categorized children with asthma into two subgroups: mild (comprising the intermittent and mild persistent categories, characterized by less frequent daytime and nocturnal symptoms) and severe (comprising the moderate and severe persistent categories, characterized by daily symptoms and more frequent nocturnal awakenings). The analysis of the alpha diversity in gut microbiota among severe asthma, mild asthma and control groups revealed no significant differences (**Figure 1**). However, the beta diversity analysis conducted using PCoA revealed a significant difference in the unweighted UniFrac but not in the weighted UniFrac (**Figure 2**).

**Figure 3:**
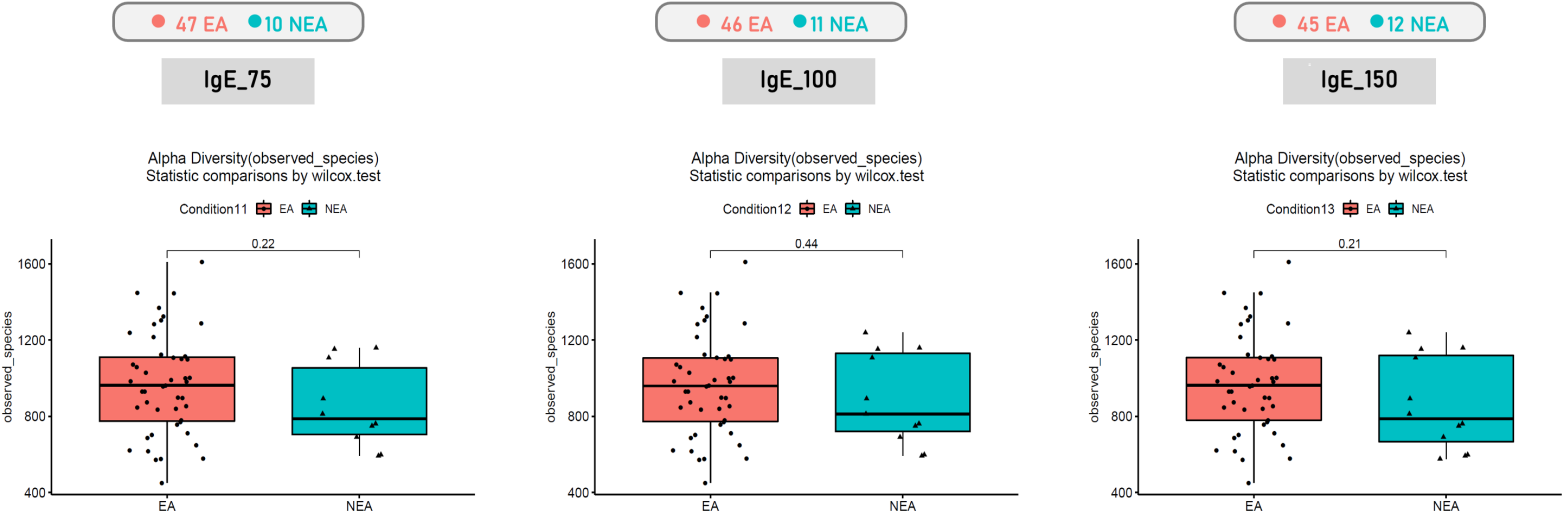
Comparison of alpha diversity between allergic asthma and non-allergic asthma based on observed richness in species based on (IgE_75, IgE_100, IgE_150 in units of measurement).

**Figure 4:**
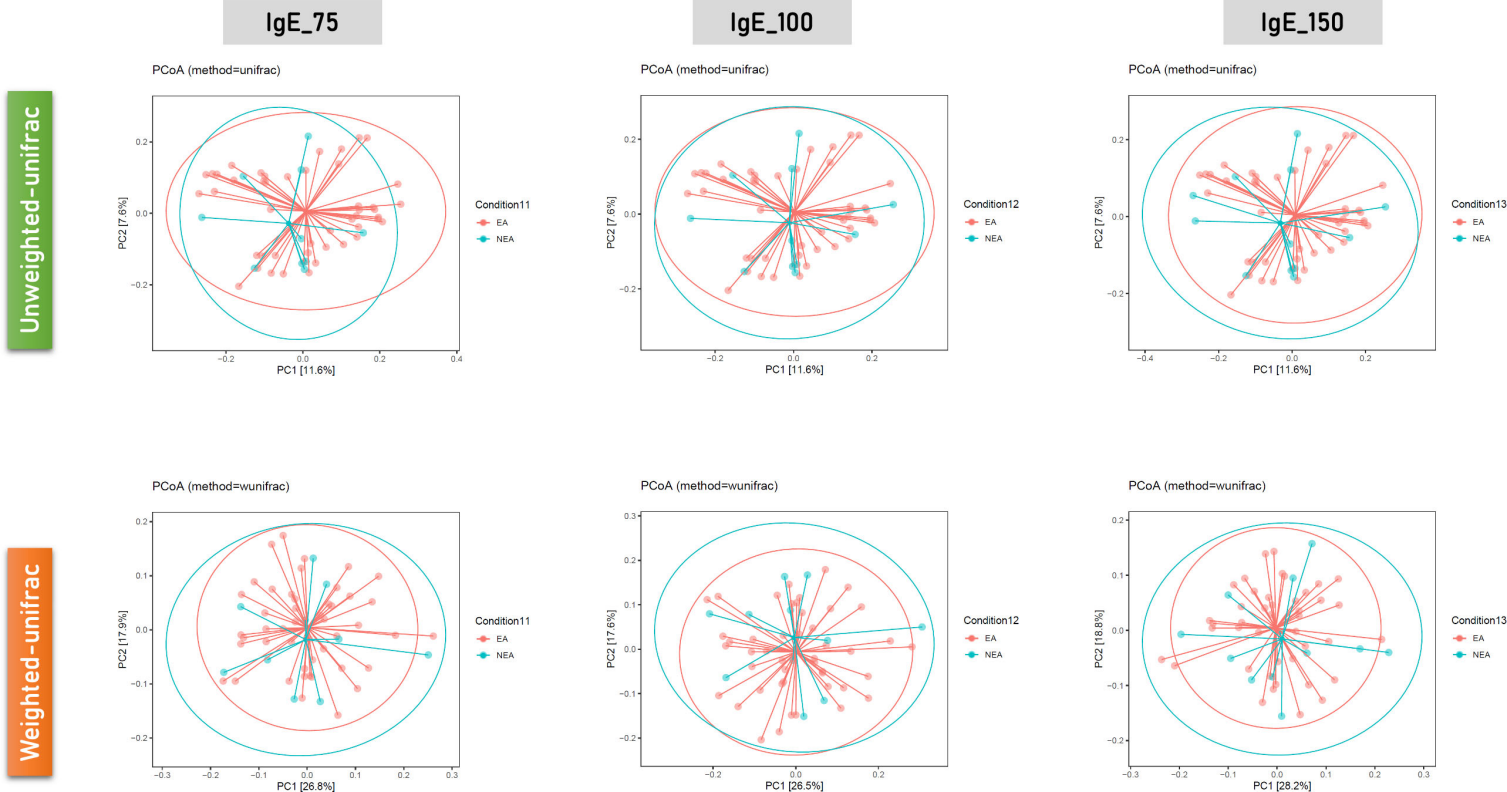
Comparison of beta diversity between allergic asthma and non-allergic asthma based on IgE levels (IgE_75, IgE_100, IgE_150 in units of measurement) conducted using principal coordinate analysis (PCoA). Upper panel, unweighted UniFrac; lower panel, weighted UniFrac.

For different phenotypes, we categorized children with asthma into two subgroups: allergic and non-allergic, according to their IgE levels. The analysis of the alpha diversity in gut microbiota between allergic asthma and non-allergic asthma revealed no significant differences (**Figure 3**). Furthermore, the beta diversity analysis conducted using PCoA also revealed no significant difference in the unweighted UniFrac and in the weighted UniFrac (**Figure 4**).

### 3.3 Taxonomic alterations associated with asthma and disease severity

**Figure 5:**
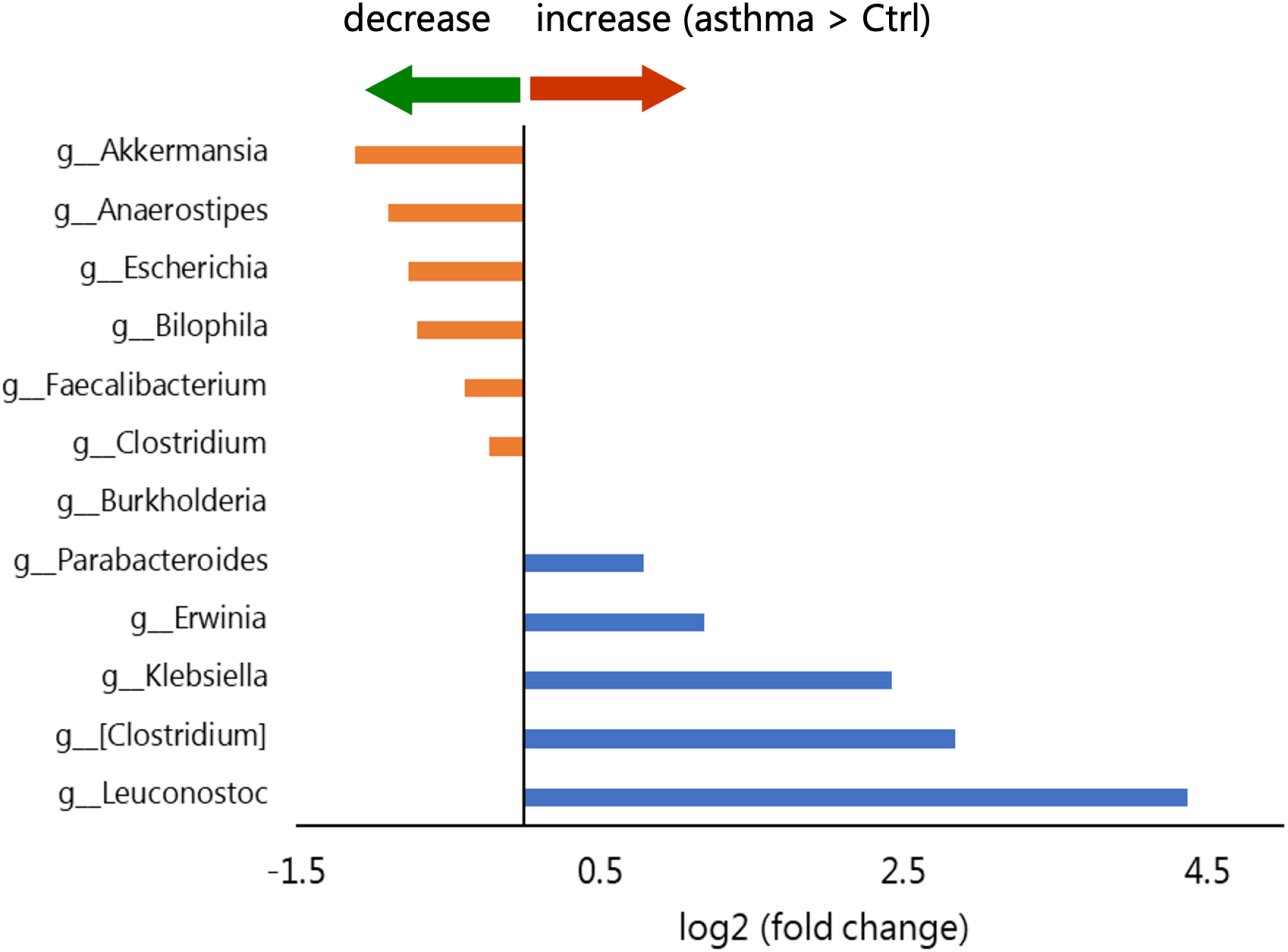
Comparison of genera selected by LEfSe based on fold change between the asthma group and the control group. Blue represents log2 (fold change) > 0 (asthma > control). Orange represents log2 (fold change) < 0 (asthma < control).

Through LEfSe, we identified a significant increase in the relative abundance of the *Erwinia*, *Klebsiella*, *[Clostridium]* (the family *Peptostreptococcaceae*) (Note: Bracketed taxa, such as *[Clostridium]*, represent polyphyletic or tentatively assigned lineages according to the Greengenes nomenclature convention.), and *Leuconostoc* genera in the children with asthma. Additionally, we noted a corresponding decrease in the relative abundance of the *Anaerostipes*, *Akkermansia*, and *Escherichia* genera (**Figure 5**).

**Figure 6:**
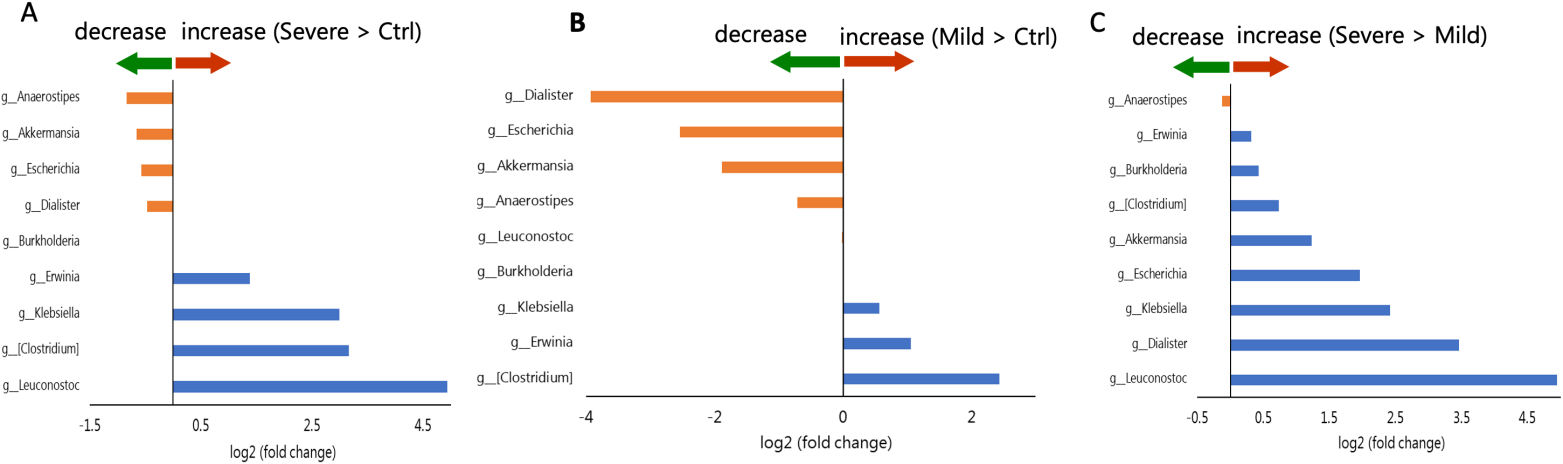
Comparison of genera selected by LEfSe based on fold change among the severe group, the mild group, and the control group. Blue represents log2 (fold change) > 0; orange represents log2 (fold change) < 0. **(A)** fold change of [mean (severe)/mean (control)] **(B)** fold change of [mean (mild)/mean (control)] **(C)** fold change of [mean (severe)/mean (mild)].

On the basis of asthma severity, LEfSe was used to compare the children in the subgroups with the control group, and the results revealed that the children in both subgroups exhibited an increase in the relative abundance of the *Klebsiella*, *Erwinia*, and *[Clostridium]* genera with a concomitant decrease in the relative abundance of the genus *Anaerostipes*. However, a decrease in the genus *Leuconostoc* was identified only in the severe subgroup (**Figure 6**).

### 3.4 Associations between specific genera and clinical asthma measurements

**Figure 7:**
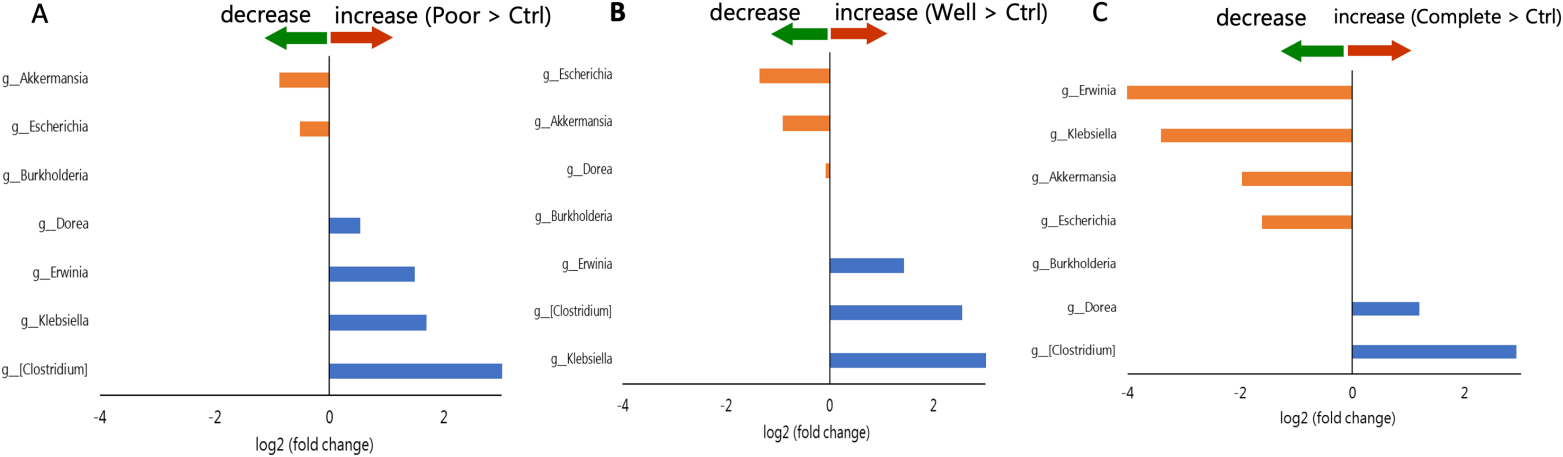
Comparison of genera selected by LEfSe based on fold change among the poor group, the well group, the complete group and the control group. Blue represents log2 (fold change) > 0. Orange represents log2 (fold change) < 0. **(A)** fold change of [mean (poor)/mean (control)] **(B)** fold change of [mean (well)/mean (control)] **(C)** fold change of [mean (complete)/mean (control)].

On the basis of C-ACT scores, we categorized the children with asthma into three subgroups: complete (C-ACT ≧ 25), well (25 > C-ACT > 19), and poor (C-ACT ≦ 19). LEfSe was used to compare the children in the subgroups with the control group, and we observed that the children with asthma exhibited an increase in the relative abundance of the genus *[Clostridium]* along with a concomitant decrease in the relative abundance of the *Escherichia* and *Akkermansia* genera, regardless of their status. Notably, as the asthma control status improved, the *Klebsiella* and *Erwinia* genera exhibited a shift from an increased abundance to a decreased abundance, with the lowest abundance noted in the complete group (**Figure 7**).

On the basis of the aforementioned findings regarding differences among severities of childhood asthma, we further conducted regression analysis for the genera *Klebsiella*, *Erwinia*, *[Clostridium]*, *Akkermansia*, and *Escherichia* to investigate whether the relative abundance of these genera was correlated with IgE levels, C-ACT scores, or peak expiratory flow (PEF). The correlation analysis showed significant differences not only in the association between the genus *Escherichia* (beta = −0.337, p = 0.016) and C-ACT scores but also between the genus *Akkermansia* (beta = −0.325, p = 0.032) and PEF.

### 3.5 Longitudinal microbial shifts between exacerbation and remission phases

In addition, we conducted longitudinal paired case comparisons within the same participants’ follow-up periods between asthma attack and remission phases, and the results also showed a higher relative abundance of *Akkermansia* during the more severe condition (**Table 2**).

**Table 2:** Gut microbiota signatures found in follow-up comparisons by LEfSe.

Biomarker	Group	LDA value	P_value
k_Bacteria;p_Proteobacteria;c_Gammaprobacteria;o_Enterobacteriales;f_Enterobacteriaceae;g_Erwinia	B1	2.685596727	0.004252019
k_Bacteria;p_Proteobacteria;c_Gammaprobacteria;o_Enterobacteriales;f_Enterobacteriaceae;g_Enterobacter	B1	2.775615420	0.041544306
k_Bacteria;p_Firmicutes;c_Clostridia;o_Clostridiales;f_Peptococcaceae;g_Peptococcus	G1	3.392532877	0.034560364
k_Bacteria;p_Verrucomicrobia;c_Verrucomicrobiae;o_Verrucomicrobiales;f_Verrucomicrobiaceae;g_Akkermansia	B1	3.661087357	0.022903557

Group G1, The better condition (remission phase) group of the same participants.

Group B1, The more severe condition (asthma attack) group of the same participants.

## 4 Discussion

In this longitudinal study, we characterize the dynamic shifts of gut microbiota in pediatric asthma. Our primary finding is that the relative abundance of *Akkermansia* spp. is not only significantly reduced in asthmatic children compared to healthy controls but also fails to recover during the remission phase. This persistent dysbiosis correlates strongly with clinical control scores (C-ACT). While previous studies have noted cross-sectional differences, our longitudinal data highlight that *Akkermansia* dynamics may serve as a key indicator of the asthmatic gut, revealing a paradoxical enrichment rather than just a transient marker of acute inflammation.

Consistent with prior research, our investigation revealed differences in the gut microbiota composition of children with asthma compared with healthy controls. Notably, an increased relative abundance of the genera *Erwinia, Klebsiella, Clostridium* (family *Peptostreptococcaceae*), and *Leuconostoc* was observed in the asthma group, along with a reduced relative abundance of *Anaerostipes, Akkermansia, and Escherichia.*

Further subgroup analyses revealed potential associations between the abundance of specific bacterial genera and asthma severity. Specifically, an increased relative abundance of the genus *[Clostridium]*, alongside a decreased relative abundance of *Akkermansia* and *Escherichia*, was consistently observed across all asthma severity levels. However, the relative abundances of *Akkermansia* and *Escherichia* were significantly lower in children with mild asthma compared with those with moderate-to-severe asthma. Moreover, children with severe asthma exhibited an increased abundance of *Leuconostoc* and a concomitant reduction in *Anaerostipes*.

Based on the above findings, this study identified three genera as potential microbial signatures for asthma: *[Clostridium]* (the family *Peptostreptococcaceae*), *Akkermansia*, and *Escherichia*. The identification of *[Clostridium]* as a candidate microbial taxon in our cohort aligns with a systematic review suggesting that elevated *Clostridium* abundance in early childhood is associated with the subsequent development of asthma (13). Mechanistically, we hypothesize that this association may be mediated by metabolic alterations, as prior studies (26–28) indicate that *Clostridium* expansion correlates with reduced fecal butyrate—a key anti-inflammatory metabolite. While direct metabolomic profiling was not performed in this study, the *[Clostridium]* signature observed here points toward a potential deficit in butyrate-mediated immune regulation, highlighting a critical pathway for future investigation.

The observed reduction in the abundance of *Akkermansia* spp. in the asthma group is consistent with findings from previous studies. Demirci et al. revealed that the abundance of *Akkermansia muciniphila* was associated with a lower risk of asthma (29). This species can potentially suppress inflammation by releasing metabolites through a pathway that enhances IL-10 production and inhibits IL-12 secretion (30). Another proposed mechanism involves the production of propionate through mucin degradation by *Akkermansia muciniphila*, which benefits both the gut ecosystem and host immune regulation (31).

In contrast, our finding of a significantly decreased relative abundance of *Escherichia* in the asthma group differs from reports in the literature. One investigation found that elevated *Escherichia* levels were associated with increased β-alanine and 4-hydroxybutyrate concentrations, alongside reduced butyrate production, and were further linked to mite allergen–specific IgE levels (32). Another study suggested that *Escherichia* spp. plays a central role in the infant gut resistome, which is positively correlated with asthma-associated bacteria and reflects the immaturity of the gut microbiota (33). This discrepancy may partly reflect differences in study populations, as most previous studies enrolled infants or preschool-aged children from Western countries, whereas our cohort comprised school-aged children of East Asian ethnicity. Variations in disease phenotype and taxonomic database methodology may also have contributed, and further cross-ethnic, multi-center studies are warranted to clarify this divergent pattern.

Although *Akkermansia* depletion characterized the general asthmatic cohort, the paradoxical relative elevation observed in the severe group compared to the mild group warrants careful interpretation. A similar link between *Akkermansia* abundance and disease severity has been noted in other allergic phenotypes, such as atopic dermatitis (34), confirming its sensitivity to inflammatory states. In our cohort, the pattern seen in severe cases likely reflects a treatment-induced alteration. Children with severe asthma are subjected to higher cumulative doses of corticosteroids. Specifically, during acute exacerbations, these patients typically received a short course of systemic oral corticosteroids (OCS) at a dose of 1 mg/kg/day for 3 days. Prior evidence indicates that corticosteroids can profoundly reshape the gut microbiome (35, 36). Corticosteroids are known to suppress T-cell activation and alter mucosal cytokine profiles (e.g., IL-10, TGF-β) (37, 38). We hypothesize that this specific immunosuppressive environment in asthma may create an ecological niche that favors the paradoxical overgrowth of *Akkermansia*, distinct from the depletion typically seen in mild, immune-active phenotypes. Thus, the observed recovery of *Akkermansia* in severe cases may be a treatment-associated shift, masking the underlying dysbiosis seen in milder, unmedicated phenotypes.

In addition, we noted an increase in *Leuconostoc* abundance accompanied by a reduction in *Anaerostipes* abundance in the severe asthma group. Anaerostipes are lactate-utilizing bacteria capable of producing butyrate, which is known to confer protection against allergic sensitization (39, 40). Conversely, *Leuconostoc*, a genus of lactic acid bacteria, has also been considered protective against allergens (41). Thus, the observed increase in *Leuconostoc* in severe asthma contradicts existing hypotheses and merits further exploration.

Notably, five genera were selected on the basis of our initial group comparison and subsequent subgroup analysis to evaluate correlations with objective asthma measurements. While previous studies have suggested associations between IgE levels and specific genera (27, 32), our analysis did not replicate these findings. Unexpectedly, we identified a negative association between C-ACT scores and the abundance of the genus *Escherichia*, as well as a negative association between PEF and the abundance of the genus *Akkermansia*. The robustness of the relationships were confirmed through paired analyses across different asthma severity status, suggesting that *Akkermansia* may represent a valuable microbial marker for assessing and monitoring asthma severity.

This study has some limitations. The taxonomic assignment was performed using the GreenGenes database. The reliance on this database may have affected the resolution of certain taxonomic classifications. Reanalysis of these sequence datasets using modern databases, such as SILVA or GTDB, is strongly recommended for future work to validate these taxonomic shifts. Additionally, fecal metabolites were not directly measured, so the functional mechanism remains to be validated. Future investigations integrating direct metabolomics or predictive functional profiling (e.g., PICRUSt2) are necessary to substantiate these hypothesized metabolic pathways. Third, without predictive modeling (e.g., ROC analyses) and independent cohort validation, the identified bacteria should be viewed as candidate microbial signatures rather than definitive biomarkers.

This study also has notable strengths. It recruited a relatively large cohort, enabling the detection of potential biases and facilitating subgroup analyses. Furthermore, participants were additionally stratified according to asthma severity, based on both C-ACT scores and clinical symptoms, revealing specific genera to be associated with C-ACT scores and PEF. Interestingly, the genera identified through LEfSe exhibited no association with serum IgE levels, highlighting distinct microbial signatures beyond atopic markers. Finally, our comparison of paired cases with different severity statuses demonstrated that the relative abundance of specific microbial genera may serve as potential indicators for disease monitoring.

## 5 Conclusions

This longitudinal study provides new insights into the non-linear relationship between gut microbiota and asthma severity. While *Akkermansia* depletion characterizes the general asthmatic state, we identified a paradoxical enrichment of this genus in children with severe, uncontrolled asthma. This severe asthma-associated gut profile, confirmed by longitudinal tracking, suggests that disease severity or high-intensity treatments (e.g., corticosteroids) may override typical dysbiosis patterns. Consequently, *Akkermansia* abundance should be interpreted with caution—not merely as a beneficial probiotic target, but potentially as a candidate microbial signature of disease severity or treatment load. Future interventions must move beyond generic supplementation to precision modulation based on clinical phenotype and medication history.

## Data Availability

The raw data supporting the conclusions of this article will be made available by the authors, without undue reservation.
